# Isolation of a SIR-like gene, SIR-T8, that is overexpressed in thyroid carcinoma cell lines and tissues

**DOI:** 10.1038/sj.bjc.6600156

**Published:** 2002-03-18

**Authors:** F de Nigris, J Cerutti, C Morelli, D Califano, L Chiariotti, G Viglietto, G Santelli, A Fusco

**Affiliations:** Servizio di Oncologia Sperimentale ‘E’ Istituto per lo Studio e la Cura dei Tumori Fondazione ‘G. Pascale’, 80131 Napoli, Italy; Centro di Endocrinologia ed Oncologia Sperimentale del CNR, c/o Dipartimento di Biologia e Patologia Cellulare e Molecolare, Università di Napoli ‘Federico II’, 80131 Napoli, Italy; Kimmel Cancer Center, Jefferson Medical College, 233S 10th Street, Philadelphia, Pennsylvania, PA 19107, USA; Dipartimento di Medicina Sperimentale e Clinica, Facoltà di Medicina e Chirurgia di Catanzaro, Università degli Studi di Catanzaro, 88100 Catanzaro, Italy

**Keywords:** thyroid, neoplasias, expression, SIR2, telomerase

## Abstract

We used subtractive library screening to identify the changes that occur in gene expression during thyroid cell neoplastic transformation. Complementary DNA from normal thyroid cells (HTC 2) was subtracted from a complementary DNA library constructed from a human thyroid papillary carcinoma cell line. The library was screened for genes upregulated in human thyroid papillary carcinoma cell line cells, and several cDNA clones were isolated. One of these clones has a sirtuin core and high homology with the human silent information regulator protein family. This clone, designated ‘SIR-T8’, was overexpressed in human thyroid carcinoma cell lines and tissues, but not in adenomas. The human SIR-T8 protein has a molecular weight of 39 kDa and is primarily located in the cytoplasm under the nuclear membrane. The SIR-T8 gene is located on chromosome 17q25-1.

*British Journal of Cancer* (2002) **86**, 917–923. DOI: 10.1038/sj/bjc/6600156 www.bjcancer.com

© 2002 Cancer Research UK

## 

Malignant thyroid carcinomas deriving from follicular cells are relatively rare in humans. They are divided into differentiated tumours, which retain both structural and functional follicular cell differentiation, and undifferentiated tumours, in which evidence of differentiation is lacking. Undifferentiated carcinomas are rare, rapidly progressive tumours. Differentiated tumours, which form up to 90% of clinically detected thyroid carcinomas, are of two main types: papillary carcinomas and follicular carcinomas. This classification was originally based on the dominant architectural pattern of the tumour; it is now based on cytology, encapsulation, distribution of metastasis and molecular biology findings (Hedinger *et al*, 1989).

Studies from our laboratory and from other groups have helped to identify the molecular events involved in thyroid carcinogenesis, i.e., activation of the RET/PTC and of the TrkA tyrosine kinase receptors in papillary carcinomas, activation of the ras genes by point mutation and PPARγ inactivation by fusion with PAX 8 in follicular carcinomas (Kroll *et al*, 2000), and mutation of the p53 gene in anaplastic tumours (Pierotti *et al*, 1996; Fusco and Santoro, 1997). However, the mechanisms that regulate the process of transformation of thyroid cells remain largely unknown. The identification of genes regulated by cell transformation, e.g. c-*myc* (Cerutti *et al*, 1996), NFκB p65 (Visconti *et al*, 1997), HMGI-C and HMGI (Y) (Chiappetta *et al*, 1995), galectin-1 (Chiariotti *et al*, 1993), thymosin β-10 (Califano *et al*, 1998), has also provided insight into the mechanisms regulating thyroid cell transformation. In a previous screening of a subtractive complementary DNA (cDNA) library resulting from a human thyroid carcinoma and normal cell line, we identified the HIP gene, which is overexpressed in malignant thyroid neoplasias (de Nigris *et al*, 1998).

Here we report the isolation of a novel gene, designated ‘SIR-T8’ because of its strong homology with human sirtuin proteins and more in general with the silent information regulator (SIR) family of proteins. The SIR proteins encompass a large number of genes that have highly conserved structural homologous sequences in organisms ranging from bacteria to man (Brachman *et al*, 1995; Frye, 1999). Among them, the protein best characterised is SIR-2, which belongs to a multigene family (Brachman *et al*, 1995; Frye, 1999; Sherman *et al*, 1999) that has been implicated in silencing, chromosome stability, recombination (Brachman *et al*, 1995) and decreased rate of ageing (Kaeberlein *et al*, 1999). This gene attracted our attention because about 85% of all primary human cancers has telomerase activity (Kim *et al*, 1994; Shay and Bacchetti, 1997, Sprung *et al*, 1999). Attempts are underway to identify clinical situations in which telomerase has diagnostic or prognostic potential and to devise *in situ* techniques to distinguish telomerase produced by normal *vs* cancer cells. Analysis of tumoral and normal thyroid tissues shows that the SIR-T8 gene is overexpressed in all differentiated and undifferentiated thyroid carcinomas cell lines and tissues examined. In contrast, its expression is weak or almost undetectable in all adenomas and normal thyroid tissues analysed.

## MATERIALS AND METHODS

### Cell lines

The human thyroid carcinoma cell lines (NIM1, TPC-1, NPA, WRO, ARO and FRO) are described elsewhere (Cerutti *et al*, 1996). They were grown in DMEM plus 10% foetal calf serum. HTC-2 cells were established and cultured as described (Curcio *et al*, 1994). The human thyroid papillary carcinoma (NPA) cell line was transfected according to the calcium-phosphate transfection protocol (Graham and van der Eb, 1973). Transfected cells were selected in a medium containing G418 (400 μg ml^−1^).

### Subtractive library construction and screening

A subtraction library was constructed enriching for NPA-specific sequences with respect to normal thyroid-specific sequences as described previously (de Nigris *et al*, 1998). Differential plaque hybridization screening of the NPA(-NT) cDNA library was performed according to a standard procedure (Sambrook *et al*, 1989). A human melanoma cDNA library constructed in Lambda gt11 vector (Stratagene, La Jolla, CA, USA) was used to isolate full-length cDNA. The library was screened according to protocols provided by the manufacturer.

### DNA sequencing and computer analysis

Positive phages from melanoma cDNA library screening were purified from the phage lysate (Sambrook *et al*, 1989) and digested with *Sal*I. Inserts were subcloned in pBluescript II SK (Stratagene) and automatically sequenced. The full-length amino acid sequences were compared with sequence of the other sirtuin using ‘Blast 2 sequences’ from the NCBI Blast website (www.ncbi.nih.gov/gorf/bl.html). The European Molecular Biology Laboratory/GenBank database was searched for known sequences.

### RNA extraction and Northern blot analysis

Total RNA was isolated from cultured cells with the guanidine thiocyanate method, slightly modified (Sambrook *et al*, 1989). Northern blot analysis, was performed according to standard procedures (Sambrook *et al*, 1989). A 300 bp cDNA fragment of the SIR-T8 gene was radiolabelled by the random primer method (Feinberg and Vogelstein, 1983). Hybridisations were carried out under stringent conditions according to standard procedures. Hybridisation signals were normalised using a glyceraldehyde-phosphate-dehydrogenase (GAPDH) probe. The filters from normal human and mouse embryo tissues were purchased from Clontech (Palo Alto, CA, USA).

### RT–PCR analysis of the SIR-T8 expression in thyroid tumours

Five micrograms of total RNA digested with RNAse-free DNAse were reverse transcribed using random exanucleotides as primers (100 mM) and 12 units AMV reverse transcriptase (Promega); 0.2 μg of cDNA were amplified in 25 μl of the reaction mixture containing 0.2 mM dNTPs, 1.5 mM MgCl_2_, 4 mM of each primer, 1 unit of Taq DNA polymerase (Perkin-Elmer) in Taq polymerase buffer. After the first denaturing step at 94°C for 2 min, PCR amplification was performed for 30 cycles (94°C for 50 s, 50°C for 50 s, 72°C for 50 s). Sequences of forward and reverse primers used were: 5′-TACCGGATCCAGCGTCTATCCAGACTAC-3′ and 5′-CTCAGCTGGACGTCCGTATTCGAAGGTA-3′. In addition to primers of the SIR-T8 gene, a set of primers designed to recognize a cDNA for the constitutively expressed enzyme GAPDH was used in each reaction as an internal control for the amount of cDNA tested. The GAPDH specific primers were: forward 5′-ACATGTTCCAATATGATTCC-3′ and reverse 5′-TGGACTCCACGACGTACTCA-3′ corresponding to nucleotides 195–215 and 355–335, respectively (Tokunaga *et al*, 1987). The amplified products were separated by 1.5% agarose gel electrophoresis and after Southern blotting, hybridised with a SIR-T8 cDNA probe.

### Construction of cDNA expression vector

The entire cDNA was digested from pBluescript using *Kpn*I/*Bgl*II, separated on 1% agarose gel, purified with a gel extraction kit (Qiagen) and subcloned into the same sites of a mammalian expression vector pFlag CMV (Stratagene). The cDNA was cloned in-frame with a Flag epitope under the transcriptional control of the cytomegalovirus promoter. This vector also carries the gene for G418 resistance.

### Immunoblotting and immunocytochemical analysis

Total protein extracts were prepared as described elsewhere (Cerutti *et al*, 1996). Western blot was revealed with monoclonal antibodies against the p/Flag epitope at 1 out of 200 dilution (Sigma Chemical Co.). NPA cells were grown on coverslips for 24 h in DMEM supplemented with 5% foetal calf serum then transfected; 24 h after transfection, the cells were briefly rinsed with PBS and fixed with 3% (w v^−1^) paraformaldehyde in PBS for 15 min at room temperature and then permeabilised with 0.2% Triton for 5 min. The coverslips were incubated with primary antibody in PBS for 1 h at 37°C. After three rinses of 5 min each with PBS, the cells were incubated with the second antibody, fluorescein-conjugated antimouse IgG (Amersham Corp.) for 40 min at 37°C. Unbound antibody was removed by several 5 min rinses at room temperature with PBS prior to preparation for fluorescence microscopy.

### Chromosome localisation

A human fibroblast genomic library in the λFIXII vector (Stratagene) was screened at high-stringency conditions using the SIR-T8 probe according to a standard procedure (Sambrook *et al*, 1989). Four phages were isolated. One was further characterised: the insert was excised and sequenced. It shows identity with the human SIR-T8 sequence. This phage was then used for FISH analysis, which was performed as described previously (Veronese *et al*, 1995; Califano *et al*, 1998).

## RESULTS

### Construction and differential screening of a cDNA subtractive library between NPA cells and normal thyroid cells

**Figure 1:**
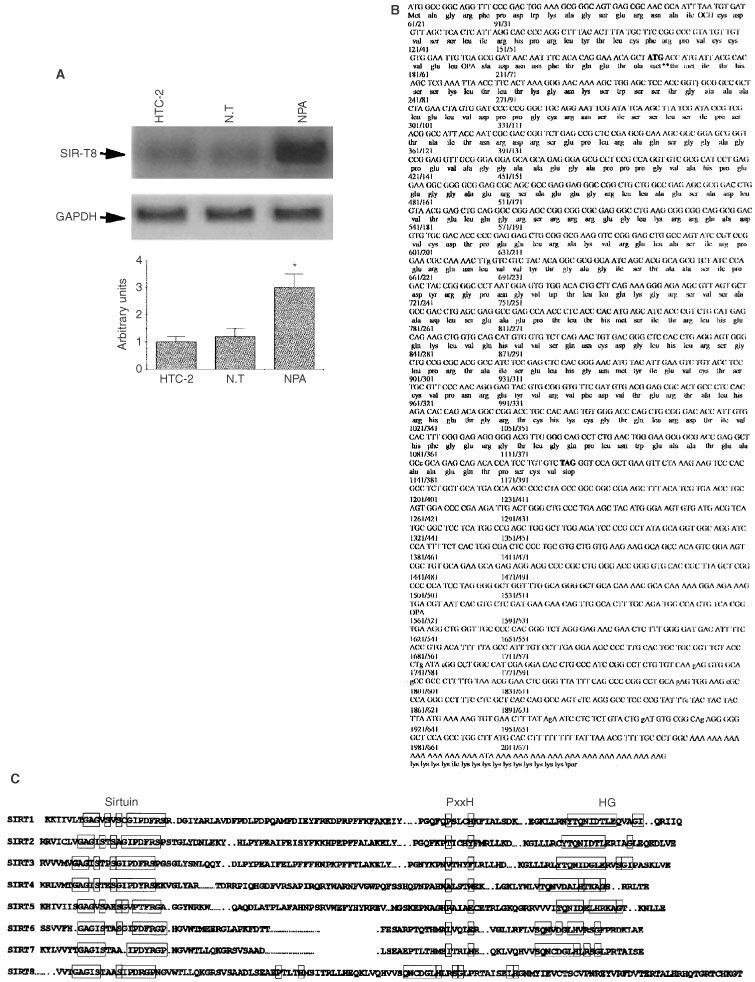
Northern blot analysis of Sir-T8 expression. (**A**) Total RNA (5 μg per lane) was hybridised with SIR-T8 probe. Lane 1: normal thyroid cell line HTC-2, lane 2: Normal tissue; and lane 3: NPA cell line. A GAPDH probe was used as internal control for uniform RNA loading. Lower part: densitometric analysis. Bars present the mean±s.d. of the results obtained in three experiments. *P<0.001 *vs* HTC-2 and normal thyroid tissue (NT). (**B**) cDNA and amino acid sequences of SIR-T8. Nucleotides are numbered from the beginning of the sequence, and the deduced amino acid sequence is indicated from the beginning of the open reading frame. An in-frame stop codon is indicated in bold. (**C**) Comparison of the amino acid sequence of SIR-T8 with other known human SIR genes; the conserved domains are boxed.

A cDNA subtractive library between the NPA cell line, established from a papillary human thyroid carcinoma, and HTC 2 cells from normal thyroid tissue, was constructed enriching the NPA-specific sequences, as previously described (de Nigris *et al*, 1998; see Materials and Methods). Subsequently, this library was screened by hybridisation of duplicated filters with cDNAs from poly(A)^+^ mRNAs from NPA and HTC 2 cells, respectively. After three consecutive screenings, we isolated 39 clones that gave rise to a much stronger signal with the NPA probe than with the HTC 2 probe. Six of these clones showed a 100% identity with a recently isolated gene, designated ‘HIP’, which encodes a heparan sulphate/heparin-binding protein. Four other clones showed a significant homology with a SIR-like protein family that encodes telomerase-related proteins (Brachman *et al*, 1995). Northern blot analysis confirmed the presence of a 2.4 kb band about three-fold stronger in NPA cells than in HTC-2 control cells and thyroid normal tissue (*P*<0.001 *vs* HTC-2 and N.T.) (Figure 1A

).

### Isolation and characterisation of a novel gene homologous to SIR family protein and designated ‘SIR-T8’

The isolated cDNA contained an incomplete open reading frame. Therefore, using the cDNA previously isolated as a probe, we screened the melanoma cell library enriched in full-length cDNA. Twelve positive clones were isolated and the inserts were sequenced (Figure 1B). The predicted amino acid sequences show a single open reading frame of 945 bp, starting with ATG codon at 162 bp and ending with a stop codon TAG at nucleotides 1110 and 874 of the untranslated 3′ end (Figure 1B). This open reading frame encodes a protein of 315 amino acid residues that has a calculated mass of 39 kDa.

A comprehensive search of the EMBL GenBank and the SWISS-Protein data base revealed that the predicted amino acid sequence was highly homologous to other known human sirtuin proteins named ‘SIR-T’ (from 1 to 7) and had an 80% identity to protein SIR-T7 (Frye, 2000). Our gene diverges from SIRT-7 in the 5′ end, but they have identical or similar amino acid residues scattered throughout the sirtuin domain. In Figure 1C all known human SIRT proteins are aligned and the conserved domain are boxed. There are several amino acidic short motifs conserved within the core domain: GAGISXXXGIPXXR, PXXXH, TQNID, HG; two sets of CXXC that may be a zinc finger domain (Brachman *et al*, 1995). The HG motif is of interest because a point mutation in this domain causes loss of sirtuin-mediated ADP-ribosylation (Frye, 1999; Tanny *et al*, 1999).

### Analysis of SIR-T8 gene expression in normal and tumoral thyroid cells

**Figure 2:**
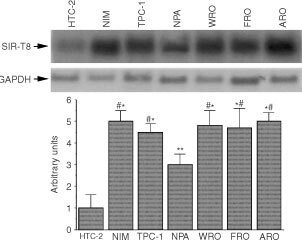
Expression of SIR-T8 in normal and tumoral thyroid cells. Total RNA (5 μg per lane) was size-fractionated on denaturing formaldehyde agarose gel, blotted onto nylon filters (hybond-N, Amersham) and probed with SIR-T8 cDNA. RNA was fractioned from the following sources: lane 1: normal thyroid primary culture cells; lane 2: NIM cell line; lane 3: TPC-1 cell line; lane 4: NPA cell line; lane 5: WRO cell line; lane 6: FRO cell line; lane 7: ARO cell line. A GAPDH probe was used as internal control for uniform RNA loading. Lower panel: densitometric analysis of SIR-T8 expression. Bars present the mean±s.d. of the results obtained in three experiments. *P<0.0001 *vs* HTC2, #*P*<0.005 *vs* NPA and **P<0.001 *vs* HTC2.

To investigate whether SIR-T8 gene overexpression is a general event in thyroid neoplastic transformation, we analysed its expression in other thyroid carcinoma cell lines of various tumour histotypes by Northern blot. The following cell lines were examined: NIM 1, NPA and TPC-1 from papillary carcinomas; WRO from a follicular carcinoma; and FRO and ARO from anaplastic carcinomas. As shown in Figure 2

, SIR-T8 expression was high in all carcinoma cell lines and almost undetectable in normal HTC-2 thyroid cells. SIR-T8 gene expression was particularly abundant in ARO, WRO and FRO cells (five-fold *vs* HTC-2 *P*<0.001) and three-fold higher in NPA than in HTC-2 *P*<0.05 (Figure 2, lower panel).

**Figure 3:**
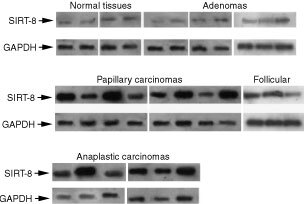
SIR-T8 expression in normal and neoplastic thyroid tissues. RT–PCR analysis of SIR-T8 in normal and neoplastic thyroid tissues. The SIR-T8 mRNA was coamplified with GAPDH as an internal control. RNA sources were divided by pathology as indicated.

Subsequently, we evaluated SIR-T8 gene expression in biopsies from eight papillary carcinomas, three follicular carcinomas, six anaplastic carcinomas and seven adenomas. SIRT-T8 expression was very weak in normal thyroid and in adenomas (Figure 3

, upper panel), and abundant in most of the human differentiated papillary and follicular thyroid carcinomas (Figure 3, middle panel) and anaplastic carcinomas (Figure 3, lower panel).

### SIR-T8 protein detection and subcellular localisation

**Figure 4:**
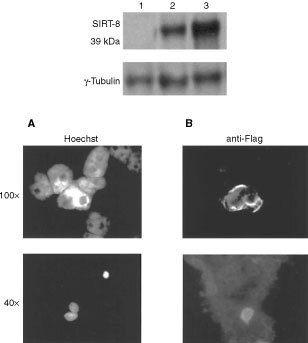
Upper panel: Western blot analysis of protein SIR-T8. Total protein extracts from the NPA cell line transfected and untransfected with p/Flag-SIRT-8 construct were subjected to Western blot analysis with antiFlag monoclonal antibody. Lane 1: untransfected NPA cells; lane 2: NPA cells transfected with the p/Flag-SIRT-8 construct; lane 3: COS cells transfected with the p/Flag-SIRT-8 construct as control. Lower panel: Subcellular localisation of SIR-T8. NPA cells were grown on coverslips for 24 h, transfected with SIR-T8 expression vector and stained with Hoechst (**A**), or immunostained with anti-Flag antibodies (**B**) as described.

To define the cellular localisation of the SIR-T8 protein, the full-length cDNA was cloned in a pFlagCMV vector. The Flag epitope identifies the recombinant protein but not the endogenous form of SIRT-8. This construct was transiently transfected in the NPA cell line, which, because of its morphology and shape, makes it easier to locate the recombinant protein. In NPA-transfected cells Western blot revealed a 39 kDa protein that was not detectable in NPA-untransfected cells (Figure 4

, upper panel)**.** NPA transfected cells were fixed with paraformaldehyde and stained with anti-Flag antibodies. There was intense cytoplasmic staining in the NPA-transfected cells (Figure 4B). At higher magnification the signal was found to be localised to the perinuclear membrane of NPA cells (Figure 4B).

### SIR-T8 gene tissue distribution and chromosomal localisation

**Figure 5:**
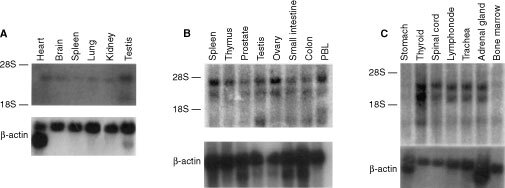
Northern blot analysis of SIR-T8 gene in different normal tissues. SIR-T8 gene expression was analysed in Poly(A)^+^ RNAs from various normal tissues as indicated.

Northern blot analysis of poly(A)^+^ mRNAs from different human tissues revealed three mRNA species: 3.5, 2.4 and 1.2 kb (Figure 5

). Gene expression was very weak in stomach and bone marrow. Also spleen, brain, lung and kidney (Figure 5A) expressed low amounts of SIR-T8. These transcripts diverge in their non-coding regions. However, normal thyroid and carcinoma cell lines preferentially express the 2.4 kb mRNA that drives 39 kDa protein synthesis.

**Figure 6:**
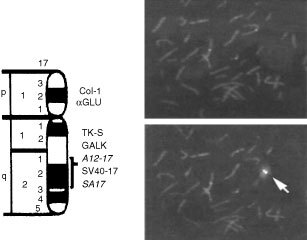
FISH mapping of the SIR-T8 gene to chromosome 17q25.1. Left ideogram of chromosome 17. Right 4′,6-diamidino2-phenylindole (top) and corresponding propidium (bottom) FISH analysis of normal lymphocyte metaphase with a phage clone the SIR-T8 gene hybridisation. Note the signal on chromosome 17q25.1.

To identify the human chromosomal location of the gene, we screened a human fibroblast genomic library with the SIR-T8 probe. Four phages were isolated. The insert of one of these phages was excised; a 3.0 kb fragment was subcloned by digestion and sequenced. Because its sequence was identical to the cDNA sequence of SIR-T8, this phage was used for FISH analysis, which showed that the SIR-T8 gene maps to chromosome 17q25-1 (Figure 6

).

## DISCUSSION

Differential screening of a subtractive library between a papillary carcinoma cell line (NPA) and normal thyroid cells (HTC-2) led to the isolation of several cDNA clones which were overexpressed in NPA cells. One of the clones corresponded to a novel gene; therefore we isolated the entire cDNA. This cDNA encodes a protein of 315 amino acids with a predicted molecular weight of 39 kDa. It is highly homologous to the sirtuin gene family that comprises seven human genes, i.e. SIR-T 1–7. Consequently, we called the gene ′SIR-T8’. Human SIR-T8 had a very high BLASTp score with 85% identity in the core sirtuin domain to the SIR-T7 protein. The SIR-T8 protein has several short motifs of conserved amino acids within the sirtuin core domain that is common to SIR protein: the GAGISTXXGIPDFR sirtuin core motif, a GVWTL motif at four residues from the C-terminus to the core motif that is characteristic of class IV sirtuin, two sets of CXXC that may be zinc finger domains and an HG motif that is strictly conserved in all sirtuin proteins and that is probably involved in ADP ribosylation (Brachman *et al*, 1995). The enzymatic function of sirtuin is not completely understood. The best characterised of the SIR protein family is SIR-2 from yeast. It mediates histone deacetylation (Tanny *et al*, 1999) thereby suggesting a coupled reciprocal activation mechanism involving interactions of SIR2 with NAD and the N-acetl-lysine groups of acetylated histones (Imai *et al*, 2000; Landry *et al*, 2000).

Here, we report that SIR-T8 codes for a protein that is primarily localised in the cytoplasm under the nuclear membrane. Conversely, most SIR proteins that have telomerase activity are in the nucleolus (Morales *et al*, 1999). However, our finding is in agreement with the subcellular localisation of other two SIR2-related proteins: SIR2L and SIR2p *Leishmania major* is absent from the nucleus (Zemzoumi *et al*, 1998).

SIR-T8 was overexpressed in seven carcinoma cell lines but weakly detectable in normal thyroid cell line and tissue. The expression of SIR-T8 was abundant in most of the human thyroid carcinomas analysed. Expression was low or absent in normal thyroid cells tissues and benign adenomas. These results suggest that SIR-T8 induction is a general event in thyroid cell transformation.

Although the function of protein SIR-T8 needs to be defined, by analogy with the Sir 2 gene, SIR-T8 gene overexpression may play a role in thyroid carcinogenesis. In fact, Sir 2 gene overexpression in yeast causes chromosome instability and a loss in cell viability (Holmes *et al*, 1997), and interactions between Sir 2 and other SIR genes or histones induce the silenced chromatin state (Ivy *et al*, 1986; Moretti *et al*, 1994). Moreover, these proteins modulate the chromatin structure, thereby maintaining patterns of gene expression throughout the development of multicellular organisms, and they are required for telomere maintenance (Orlando and Paro, 1995). Telomeres are specialised structures at the ends of eukaryotic chromosomes. They are ribonucleoproteins that synthesize telomeric DNA on the chromosomal end using a segment of its RNA as template. In most types of human somatic cells the telomeres shorten as with organism age and with repeated passages in culture. The immortal cells that elude crisis are characterised by stable levels of telomerase activity and by stable telomeres. This suggests that telomerase activation can overcome the limitation imposed by telomere length on the lifespan of cell lineages (Counter *et al*, 1992). In humans, telomerase activity is detectable in germline cells and in certain stem cells, but not detectable in most somatic cell lineages (Harley *et al*, 1997). It is also detected in most human tumour samples analysed (Counter *et al*, 1994; Kim *et al*, 1994) suggesting that telomerase activation may represent an essential step in tumour progression. Moreover, increased expression of the other telomerase proteins correlate with the metastatic phenotype (Sprung *et al*, 1999).

In conclusion, although the mechanism underlying enhanced SIR-T8 gene expression in thyroid tumours is unknown, the homology of SIR-T8 with the telomerase proteins, and its similarity with telomerase proteins suggests its increased expression might have a role in the process of thyroid carcinogenesis.
