# Isolation of a SIR-like gene, SIR-T8, that is overexpressed in thyroid carcinoma cell lines and tissues

**DOI:** 10.1038/sj.bjc.6600636

**Published:** 2002-11-26

**Authors:** F de Nigris, J Cerutti, C Morelli, D Califano, L Chiariotti, G Viglietto, G Santelli, A Fusco

## Abstract

*British Journal of Cancer* (2002) **87**, 1479–1479. doi:10.1038/sj.bjc.6600636 www.bjcancer.com

© 2002 Cancer Research UK

## 

**Correction to:** *British Journal of Cancer* (2002) **86**, 917–923. doi: 10.1038/sj.bjc.6600156

We have reviewed our data about the cloning of the novel putative SIR-like gene and we accept Dr Frye's assertions (Frye, 2002). We are grateful to him for his corrections. Therefore, we retract the claim for a novel SIR-like gene, SIR-T8. We apologize to the scientific community for the misinterpretation of our data. However, we would like to point out that the main point of our article, that is the finding that SIR-T7 is overexpressed in human thyroid carcinoma cell lines and fresh biopsies, remains valid.
